# Write It

**Published:** 1878-05

**Authors:** 


*Write It.*—Why not? You know facts in your practice that are of use to you, which you have reason to think the public are not generally familiar with. You are studying hard, are a close observer, and are original to some extent at least in your methods of work. Pathological anomalies and surgical cases occasionally fall under your observation, and you are certain that your individuality has struck out a path which has led to good results. Write this out, friend, and send it to the JOURNAL. If you cannot give it that graceful shape and elegant style which characterizes the better productions of the day, put it down anyhow. If, as a friend said a few days ago, you cannot "make it elegant and have it strong, too," let us have the facts. The editors are willing, (so industrious souls are they,) to rewrite anything that has good solid *facts* in it, if you will only give those facts. We will try and put it in shape if need be. So write it.

